# RECONSTRUCTING PERSON-CENTEREDNESS FROM A SYSTEMS PERSPECTIVE: IMPLICATIONS FOR THE CARE OF OLDER ADULTS

**DOI:** 10.1093/geroni/igad104.3556

**Published:** 2023-12-21

**Authors:** Ayesha Syed, Philip Sloane, Sheryl Zimmerman, Sam Fazio

**Affiliations:** University of North Carolina at Chapel Hill, Chapel Hill, North Carolina, United States; University of North Carolina at Chapel Hill, Chapel Hill, North Carolina, United States; University of North Carolina at Chapel Hill, Chapel Hill, North Carolina, United States; Alzheimer’s Association, Chicago, Illinois, United States

## Abstract

Person-centeredness has become a common, almost hackneyed phrase in the context of care for older adults, despite a lack of consensus regarding its definition, measurement, and application. As part of a series of studies aimed at better understanding person-centeredness, we sought to identify key themes about person-centeredness as it applies to the system (macro) level, as opposed to the setting (meso) and interpersonal (micro) levels. Four think-tank meetings (two in-person; two virtual) were held and results analyzed; the 32 participants included researchers, providers, policy makers, ethicists, and consumers. Think-tank meeting prompts addressed a range of systems, including education, government, workplace, and healthcare. Analyses identified themes associated with variation in person-centeredness. Five key themes emerged: (1) The goals and manifestation of person-centeredness vary depending on the type of system, level within a system, and the role of the individual within the system. (2) Principles of person-centeredness from the hospitality industry can be applied to other settings, including healthcare and long-term care. (3) Some limits on person-centeredness are necessary for system success. (4) The amount of person-centeredness provided in a setting depends on available resources such as time and money. (5) Societal inequities, such as discrimination, bias, and language barriers, hinder person-centeredness. Presentation of results will draw parallels between a variety of systems, demonstrating universal applicability, including to healthcare and long-term care for older adults. The themes identified may help guide future policy and practice regarding realistic and effective application and evaluation of person-centeredness in relation to the care of older adults.

